# Process relevant screening of cellulolytic organisms for consolidated bioprocessing

**DOI:** 10.1186/s13068-017-0790-4

**Published:** 2017-04-24

**Authors:** Elena Antonov, Ivan Schlembach, Lars Regestein, Miriam A. Rosenbaum, Jochen Büchs

**Affiliations:** 1 AVT‑Biochemical Engineering, RWTH Aachen University, Forckenbeckstr. 51, 52074 Aachen, Germany; 2 Institute of Applied Microbiology, RWTH Aachen University, Worringerweg 1, 52074 Aachen, Germany

**Keywords:** Consolidated bioprocessing, Respiration activity, Cellulase activity, Cellulose, Freeze assay, Itaconic acid, *Trichoderma reesei*, *Penicillium verruculosum*

## Abstract

**Background:**

Although the biocatalytic conversion of cellulosic biomass could replace fossil oil for the production of various compounds, it is often not economically viable due to the high costs of cellulolytic enzymes. One possibility to reduce costs is consolidated bioprocessing (CBP), integrating cellulase production, hydrolysis of cellulose, and the fermentation of the released sugars to the desired product into one process step. To establish such a process, the most suitable cellulase-producing organism has to be identified. Thereby, it is crucial to evaluate the candidates under target process conditions. In this work, the chosen model process was the conversion of cellulose to the platform chemical itaconic acid by a mixed culture of a cellulolytic fungus with *Aspergillus terreus* as itaconic acid producer. Various cellulase producers were analyzed by the introduced freeze assay that measures the initial carbon release rate, quantifying initial cellulase activity under target process conditions. Promising candidates were then characterized online by monitoring their respiration activity metabolizing cellulose to assess the growth and enzyme production dynamics.

**Results:**

The screening of five different cellulase producers with the freeze assay identified *Trichoderma* *reesei* and *Penicillium* *verruculosum* as most promising. The measurement of the respiration activity revealed a retarded induction of cellulase production for *P.* *verruculosum* but a similar cellulase production rate afterwards, compared to *T.* *reesei*. The freeze assay measurement depicted that *P.* *verruculosum* reaches the highest initial carbon release rate among all investigated cellulase producers. After a modification of the cultivation procedure, these results were confirmed by the respiration activity measurement. To compare both methods, a correlation between the measured respiration activity and the initial carbon release rate of the freeze assay was introduced. The analysis revealed that the different initial enzyme/cellulose ratios as well as a discrepancy in cellulose digestibility are the main differences between the two approaches.

**Conclusions:**

With two complementary methods to quantify cellulase activity and the dynamics of cellulase production for CBP applications, *T.* *reesei* and *P.* *verruculosum* were identified as compatible candidates for the chosen model process. The presented methods can easily be adapted to screen for suitable cellulose degrading organisms for various other applications.

**Electronic supplementary material:**

The online version of this article (doi:10.1186/s13068-017-0790-4) contains supplementary material, which is available to authorized users.

## Background

Cellulose is a renewable feedstock and could replace fossil oil for the production of various compounds [1]. One concept to convert cellulose into soluble sugars is enzymatic hydrolysis. The main economical challenge, thereby, is a cheap production of cellulolytic enzymes [2]. A promising strategy to reduce costs is consolidated bioprocessing (CBP). Instead of separately producing cellulases, hydrolyzing the cellulose, and converting the released sugars into the desired product, all three processes are conducted simultaneously in one step [3]. This concept has the large advantage in that inherent product inhibition of the cellulases by their hydrolysis products is completely avoided. If no organism is found that can produce cellulases and the desired product at once, a mixed culture of microorganisms can be applied. Hence, the process cannot be optimized for each task separately but the optimum for the combined reactions must be found. This includes the identification of the most suitable cellulase-producing organisms whose cellulases have high cellulolytic potential under the desired process conditions.

Cellulase activity is most commonly expressed in filter paper units, which can only be measured in the cell-free supernatant using filter paper as substrate [4]. The standard filter paper assay, combined with the determination of the protein content, quantifies the volumetric and specific enzyme activity under optimal hydrolysis conditions (pH 4.8, temperature = 50 °C). These are important parameters to evaluate cellulase producers for separate hydrolysis and fermentation applications. Different factors have to be considered when evaluating cellulase producers for CBP. During CBP, the cellulase-producing organism is in direct contact with the cellulose material. Therefore, enzymes bound to the organism or the substrate can contribute to cellulose hydrolysis [5]. Furthermore, the fermentation conditions for the cellulase production and for the formation of the target product (e.g., itaconic acid) might deviate from the optimal hydrolysis conditions [6, 7]. Therefore, cellulase activity has to be evaluated under target process conditions instead of the optimum hydrolysis conditions.

The type of cellulose influences the enzyme production rate as well as the hydrolysis rate and should be as similar as possible or even identical to the target feedstock [8, 9]. Moreover, the hydrolysis rate decreases with the time of conversion [10, 11]. Therefore, the best way to take all of these interactions into account is to measure the in situ hydrolysis rate directly during the fermentation.

In a system consisting of filamentous fungi tightly attached to the insoluble cellulose material, instantly consuming the released sugars, the in situ hydrolysis rate is hard to measure. As shown recently for *Trichoderma reesei* Rut-C30, online measurements of the oxygen transfer rate (OTR) during cultivation on cellulose can be used to estimate the in situ hydrolysis rate [12]. However, because the screening is performed in batch in shake flasks under dynamic conditions, the pH of the cultivation broth and the concentration of cellulose changes in the course of the fermentation. These dynamic conditions make it difficult to extrapolate the results of such experiments to a specific target scenario (e.g. fixed pH). Therefore, a new so called “freeze assay” was developed with the aim to measure the cellulolytic activities under defined process conditions while still mimicking the in situ fermentation conditions as closely as possible.

The overall objective of the study was to establish new methods to (a) evaluate the maximum hydrolysis activity under targeted CBP process conditions and (b) characterize the kinetics of the in situ cellulase activity during growth and enzyme production. *T. reesei* Rut-C30, which is the most common cellulase producer, is compared with four alternative cellulase-producing fungi *Aspergillus terreus*, *Penicillium verruculosum*, *Myceliophtora thermophil*a, and *Thielavia terrestris*.

## Results and discussion

### Selection of candidate organisms for the CBP scenario: Itaconic acid production from cellulose

Due to the combination of different processes, a consolidated approach imposes very specific requirements for the operating window. The target process, here, is the direct production of itaconic acid from cellulose by a microbial mixed culture of a cellulase producer with *A.* *terreus*. Itaconic acid is an overflow metabolite of the latter, produced under glucose-unlimited conditions. As a consequence, the accumulation of glucose is a prerequisite for the process [7]. Thus, cellulase-producing organisms resistant to feedback inhibition by glucose are desirable. Itaconic acid production is favorable at an acidic pH of 3.1 or lower [6, 13]. However, a recent study of Hevekerl et al. [14] demonstrated that after the initiation of itaconic acid production, it is beneficial to increase the pH set point. Most cellulases have their optimum around pH 5 [15]. Therefore, a pH value of 3.7 was chosen as a compromise for itaconic acid formation based on cellulose. Regarding process temperature, itaconic acid production is possible between 30 and 40 °C [16, 17]. The potential cellulolytic co-culture partner should, therefore, either produce cellulases with a lower pH optimum or be capable to compensate the decrease in cellulase activity at low pH. This could be achieved by the production of higher amounts of cellulases or allowing higher fermentation temperatures that would accelerate hydrolysis rates.

With respect to these requirements, four potential co-culture partners for *A.* *terreus* were selected for the screening as listed in Table 1. *T.* *reesei* Rut-C30 was chosen as benchmark organism [18]. *A.* *terreus*, which is itself a known producer of cellulases, was investigated to compare its own endogenous cellulase activity against the cellulolytic activity of the potential mixed culture partner [19]. *P.* *verruculosum* was recently mutagenized into a promising cellulase hyperproducer with a lower pH optimum and is less prone to cellulase inhibition by residual lignin (Punt, Leiden University, the Netherlands, personal communication) [20]. *M.* *thermophila* and *T.* *terrestris* are thermophilic organisms able to grow above 45 °C and are reported to produce cellulase mixtures with superior specific activity compared to *T.* *reesei* [21, 22]. Furthermore, they produce a number of recently identified GH61-type cellulases that unlike classical cellulases cleave cellulose chains by oxidation rather than hydrolysis. These enzymes were shown to synergistically boost the activity of hydrolytic cellulases by making recalcitrant parts of the cellulose more accessible [23].

**Table 1:** Cellulase activity optima of the investigated candidate organisms and targeted consolidated bioprocessing (CBP) conditions

Fungal strain	Collection	Cellulase activity optimum		Comment	Source
		pH	Temperature (°C)		
*Trichoderma reesei* Rut-C30	ATCC 56765	4.5–5.0	50	Benchmark organism	[24, 25]
*Aspergillus terreus*	DSM 23081	5.5	55	Itaconic acid producer	[19]
*Penicillium verruculosum* M28-10	DSM 8069	2.5–4.0	45–50	Low pH active, low lignin binding	Punt, personal communication [20]
*Myceliophtora thermophila*	DSM 1799	4.8–5.5	60	Thermophilic	[21]
*Thielavia terrestris*	CBS 351.90	4.8	60	Thermophilic	[21]
Target CBP conditions^a^		3.7	30–37		

^a^Target process conditions for the mixed culture approach to produce itaconic acid from cellulose using *A. terreus* as itaconic acid producer. Process conditions were determined by growth and itaconic acid production characteristics of *A. terreus*

### General assessment of cellulase-producing candidates at different cultivation conditions

**Fig. 1:**
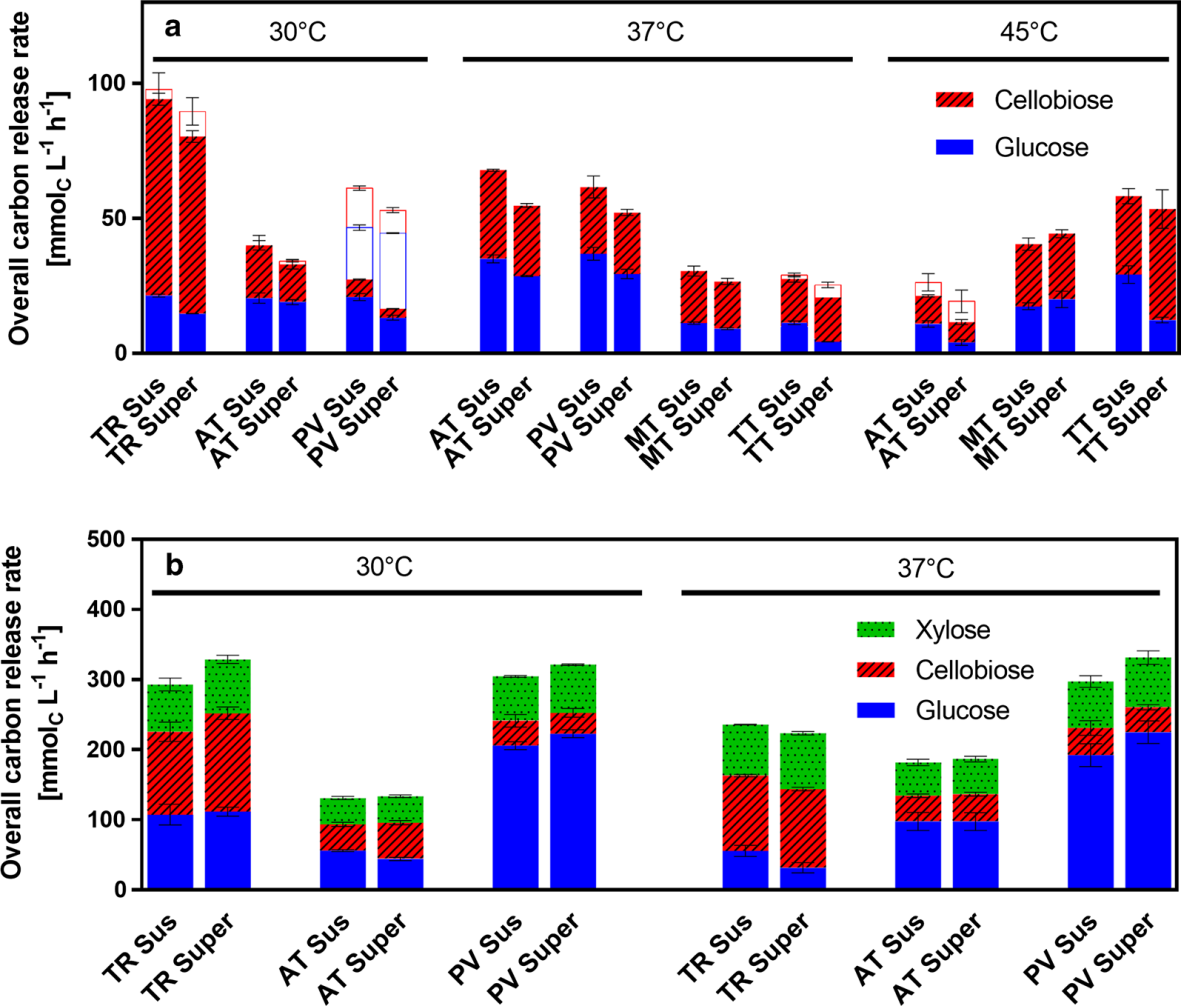
Cellulase activity screening of five candidate organisms cultivated under different conditions evaluated by the freeze assay. Freeze assay was performed at the indicated cultivation temperature with either suspended full culture broth (“sus,” first stacked bar) or supernatant (“super,” second stacked bar). *Bars* show overall carbon release rate as a sum of glucose, cellobiose and xylose carbon release rate. *Colors* indicate the distribution of these sugars. *Error bars* show standard deviation of biological triplicates. TR, *Trichoderma reesei*; AT, *Aspergillus terreus*; PV, *Penicillium verruculosum;* MT, *Myceliophtora thermophila*; TT, *Thielavia terrestris*; **a** Preliminary cellulase activity screening at a cultivation temperature of 30, 37, and 45 °C: cellulase activity was measured after 3 days of cultivation in non-buffered medium with 7.5 g L^−1^ avicel. Freeze assay was performed with avicel measuring glucose and cellobiose. *Ghost bars* show activity values measured after 10 days of cultivation in cases where maximum activity was not reached after 3 days. **b** Refined cellulase activity screening at a cultivation temperature of 30 and 37 °C: cellulase activity was measured after 5 days of cultivation in 0.1 M PIPPS buffered medium with 5 g L^−1^ glucose and 30 g L^−1^ α-cellulose. Freeze assay was performed with α-cellulose measuring glucose, cellobiose, and xylose. Assay conditions: 91 mM itaconic acid buffer (pH 3.7), 120 g L^−1^ cellulose, incubation time 2 h, filling volume 1.1 in 2-mL test tube, shaking frequency 900 rpm, shaking diameter 3 mm

A preliminary screening was performed to evaluate the cellulase producers based on conditions that were widely used for the screening of cellulolytic fungi [26, 27]. Thereby, an unbuffered medium with 7.5 g L^−1^ avicel cellulose as sole carbon source was used. The fungi were grown at different temperatures corresponding to their thermal preferences and otherwise identical growth conditions. Samples were taken after different time intervals and then evaluated for cellulase activity by the freeze assay at the corresponding growth temperature. To perform the freeze assay, a sample was mixed with additional 120 g L^−1^ cellulose and supplemented with itaconic acid buffer (pH 3.7) to establish the target process conditions. The sample is frozen to inhibit the metabolic activity (i.e., glucose consumption) of the fungus and thawed before incubating it at the fermentation temperature for 2 h. To evaluate the release of different sugar constituents from the cellulose in a comparable way, the freeze assay-based overall carbon release rate (CRR_Freeze_) was calculated using the molar amount of carbon released from cellulose in the form of glucose and cellobiose measured by HPLC. In order to localize the cellulase activity, the freeze assay was performed with either suspended full culture broth or culture supernatant. As depicted in Fig. 1, for most fungi a trend for higher activity of the culture broth than of the corresponding supernatant is observed, indicating that cell or cellulose bound cellulases contribute to the hydrolytic activity. This effect is already well known for *T.* *reesei* [28–30].

According to Fig. 1a, most of the candidates reached the maximum activity after 3 days of cultivation, except for *P.* *verruculosum* grown at 30 °C, whose activity continuously increased during the cultivation period of 10 days. Although the chosen pH condition in the freeze assay was not optimal for *T.* *reesei* enzymes, *T. reesei* reached by far the highest CRR_Freeze_ at 30 °C. Furthermore, a pronounced β-glucosidase deficiency was identified for *T.* *reesei* as can be deducted from the high cellobiose concentration. It is known that cellobiose is even a stronger inhibitor of cellulases than glucose, which strongly influences cellulase activity at high residual sugar concentrations needed for efficient itaconic acid production by *A.* *terreus* [31]. Furthermore, it was reported that the itaconic acid yield from cellobiose is slightly less than from glucose [32]. *P.* *verruculosum* showed the best β-glucosidase activity, having the smallest fraction of cellobiose in the CRR_Freeze_. This makes *P.* *verruculosum* an interesting candidate, even though the CRR_Freeze_ was lower than for *T.* *reesei.*

*Trichoderma reesei* is reported to produce much less cellulases at 37 °C than at 30 °C. Therefore, it was not investigated at 37 °C in the preliminary screening (Fig. 1a) [33, 34]. *P.* *verruculosum* reached similar CRR_Freeze_ at 30 and 37 °C after 5 or 3 days of cultivation, respectively. In contrast, a marked increase in freeze assay activity was achieved for *A.* *terreus* when cultivated at 37 °C in comparison to 30 or 45 °C. The thermophiles *T.* *terrestris* and *M.* *thermophila* clearly profited from higher growth temperatures exhibiting low activity when grown at 37 °C and increased activity at 45 °C. However, because their CRR_Freeze_ at 45 °C was still lower than the activity of *T.* *reesei* at 30 °C, the use of thermophilic conditions appears not to be beneficial for the target process. Still, the thermophilic organisms might be useful for processes, where temperatures over 37 °C are required. Strain development may, of course, change the picture significantly.

*Trichoderma reesei* and *P.* *verruculosum* were selected as the most promising cellulase producers for the selected CBP process in the preliminary screening, based on the highest total CRR_Freeze_ and the high β-glucosidase activity, respectively. For the refined screening, the best candidates along with *A.* *terreus* were tested under conditions more relevant for production at a higher cellulose concentration of 30 g L^−1^. To prevent an excessive pH drop at higher cellulose concentration, the non-metabolizable PIPPS buffer was added to the medium. As carbon source, α-cellulose was chosen, since it has been shown to have similar degradation characteristics as alkaline-pretreated biomass used in biorefineries [35, 36]. α-Cellulose contains a hemicellulose fraction and, therefore, also xylose was measured in the freeze assay [36].

In comparison to the preliminary screening, a more than twofold higher CRR_Freeze_ was achieved in all cases (Fig. 1b). As illustrated in the last section of the results, CRR_Freeze_ is a logarithmic function of the enzyme/cellulose ratio. Thus, the amount of enzyme produced under the refined conditions must have been several folds higher than in the preliminary screening, which can be attributed to the higher cellulose concentration and the more stable pH.

Nearly the same amount of xylose was detected in all freeze assay samples. Therefore, all three organisms showed a comparable xylanolytic activity.

All three fungi were grown at 30 and 37 °C to compare both temperature scenarios. At 30 °C, *T.* *reesei* and *P.* *verruculosum* had almost identical activities, whereby *P. verruculosum* again showed far less cellobiose accumulation than *T. reesei*. Like in the preliminary screening, *P*. *verruculosum* reached very similar activity at 30 and 37 °C. However, for *T. reesei* the CRR_Freeze_ clearly dropped when cultivated at 37 °C compared to 30 °C, as expected from the literature [33, 34]. Also in the refined screening, *A. terreus* showed an increase in activity when cultivated at 37 °C.

The data presented above suggest that a co-culture of *A. terreus*/*P. verruculosum* at 37 °C could result in high CRR_Freeze_. It is known from the literature that cellulase mixtures from different organisms can show synergism, resulting in higher combined activity than the sum of each individual activity [37]. However, as depicted in Additional file 1: Figure S1, in the mixture of *T.* *reesei* or *P.* *verruculosum* with *A.* *terreus* culture broths, the CRR_Freeze_ was similar and not increased compared to the unmixed sample of *T.* *reesei* or *P.* *verruculosum*, respectively. Furthermore, the performance in this case was similar at 30 and 37 °C. Thus, both *T. reesei* and *P. verruculosum* are interesting candidates for the mixed culture process.

### Characterization of most promising consolidated bioprocessing candidates

**Fig. 2:**
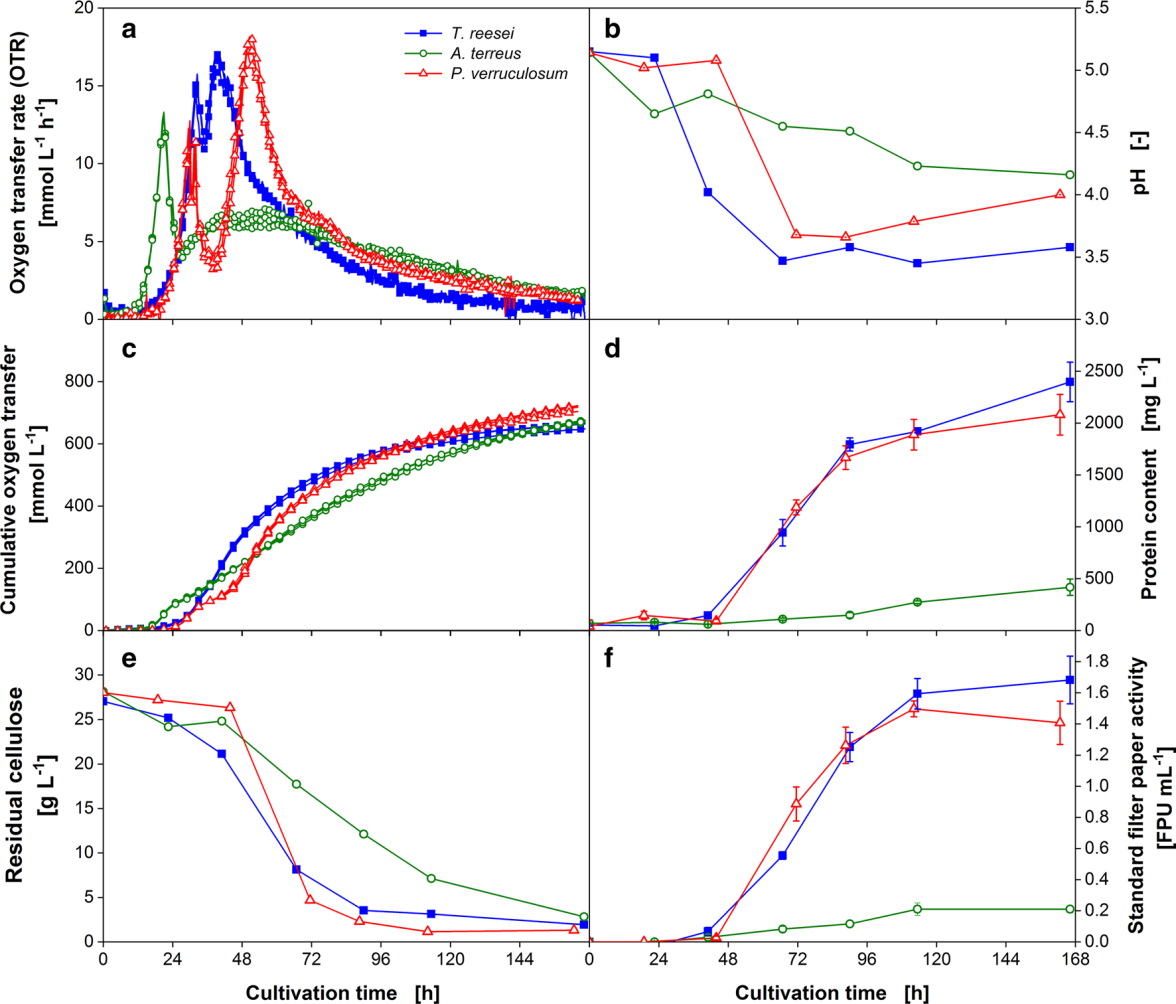
Characteristic growth and enzyme production properties of *T.* *reesei, A.* *terreus*, and *P.* *verruculosum*. **a** Biological triplicates of oxygen transfer rate (OTR) and **c** cumulative oxygen transfer. For clarity of the depicted values, only every third measuring point of OTR and every eighth measuring point of cumulative oxygen transfer over time are represented by a symbol. **b** pH value; **d** Protein content in the culture supernatant; **e** Residual cellulose concentration; **f** Standard filter paper activity; RAMOS flasks were cultivated for OTR assessment without interruption. For offline analysis in **b**, **d**–**f**, shake flasks run in parallel, inoculated from the same master mix, were harvested. *Error bars* represent standard deviation of technical triplicates from pooled biological duplicates. Culture conditions: modified Pakula medium with 5 g L^−1^ glucose and 30 g L^−1^ α-cellulose, 250-mL flask, filling volume 20 mL, shaking frequency 350 rpm, shaking diameter 50 mm, inoculum 10^6^ spores mL^−1^, and 30 °C

In the cellulase activity screening using the freeze assay performed under target process conditions, *T.* *reesei* Rut-C30 and *P. verruculosum* M28-10 displayed the highest CRR_Freeze_. Therefore, these two most promising cellulase producers and *A.* *terreus* as itaconic acid producer were characterized in detail regarding their growth and enzyme production properties. The results are presented in Fig. 2. The same cultivation conditions as in the refined cellulase activity screening were applied, except for inoculating the cultures with spores to identify the differences in germination time. As nearly the same CRR_Freeze_ was measured for the cultivation of *T.* *reesei* and *P.* *verruculosum* at 30 and 37 °C, the parameter was not crucial and the lower temperature was chosen for the process. To assess the growth and enzyme production of the fungi on the solid substrate cellulose in shake flasks, the respiration activity was measured, using the respiration activity monitoring system (RAMOS). The method was previously established to evaluate the digestibility of different types of cellulose materials by *T.* *reesei* Rut-C30 cultures [12].

Figure 2a depicts the oxygen transfer rate (OTR) of *T.* *reesei*, *A.* *terreus*, and *P.* *verruculosum* over cultivation time after inoculating the medium with 10^6^ spores mL^−1^ of the corresponding organism. The respiration activity measurement for each organism was performed as biological triplicate, and the low standard deviation of the OTR demonstrates the high repeatability of the growth in one parallel experiment. The cultures of all three organisms showed a two-peak OTR pattern varying in the height and shape of their second maximum. The mechanism behind this pattern was in detail investigated by Antonov et al. [12]. The analysis of the OTR of *T.* *reesei* Rut-C30 revealed two distinct phases during growth on cellulose. During the second increase in OTR, the easily digestible cellulose is degraded and the amount of enzymes available limits cellulose hydrolysis until the maximum OTR is reached. Then, during the following gradual drop in OTR, the amount of cellulose binding sites and digestibility of the cellulose become the limiting factors. Thus, the slope of the linear OTR increase correlates to the enzyme production rate and is an important parameter to evaluate the enzyme production at the beginning, while the maximum OTR marks the inflection point between enzyme-limited and substrate binding site-limited hydrolysis phase.

The organisms, *T. reesei* and *P.* *verruculosum,* one of which should become the cellulase producer in the target CBP application, possessed a similar lag phase of around 18 h. After the germination of the spores, the OTR increased exponentially and peaked at 15 mmol L^−1^ h^−1^ after 32 h for *T.* *reesei* and 13 mmol L^−1^ h^−1^ after 30 h for *P.* *verruculosum*. This first drop in OTR could be attributed to the exhaustion of the easy to metabolize carbon source glucose as revealed by HPLC analysis (data not shown). Glucose was included into the culture medium to shorten the lag phase of the fungi and to investigate the time needed to induce cellulase production. Thereafter, the OTR profiles deviate from each other. The OTR of *T*. *reesei* only dropped slightly to 11 mmol L^−1^ h^−1^ followed by a linear increase reaching the second maximum of 17 mmol L^−1^ h^−1^ after 40 h of cultivation. In contrast, the OTR curve of *P.* *verruculosum* exhibited a pronounced minimum of 4 mmol L^−1^ h^−1^. Subsequently, the OTR increased with a similar slope compared to the *T. reesei* culture and reached a slightly higher OTR maximum of 18 mmol L^−1^ h^−1^ after 50 h. The following gradual and slow decrease in OTR proceeded quite alike in both cultures. For the culture of *A. terreus*, a shorter lag phase of ~12 h was observed. Therefore, the first peak was reached earlier, after 20 h of cultivation. The drop in OTR was comparable to the *P. verruculosum* culture, but the second OTR maximum was very broad and flat, reaching only 7 mmol L^−1^ h^−1^.

For all cultures, the second increase in OTR coincides with the start in cellulose digestion as illustrated in Fig. 2e. Thus, it is likely that the period of time between the first OTR maximum and the following OTR increase can be attributed to the time needed to induce cellulase production. This hypothesis is supported by earlier experiments with *T.* *reesei* Rut-C30 applying cellulase-inducing compounds, which result in an earlier increase in OTR [12]. The digestion of cellulose sets in later for the *P.* *verruculosum* culture but is slightly faster compared to *T.* *reesei*. The *A.* *terreus* culture showed the slowest decline of cellulose concentration. Remarkably, at the end of cultivation nearly the same amount of cellulose is consumed in all cultures, despite *A.* *terreus* showing a sevenfold lower standard filter paper activity measured under optimal conditions for cellulases at pH 4.8 and a temperature of 50 °C (Fig. 2f). This observation reveals that the in situ hydrolysis rate must be affected predominantly by other factors than the enzyme concentration. This was also supported by the results gained during a cultivation of *T.* *terrestris,* which are shown in Additional file 2: Figure S2, but are not further relevant for the here considered CBP (and are therefore not further discussed).

The differences in the rate of cellulose digestion can also be detected on the basis of the cumulative oxygen transfer shown in Fig. 2c. The increase of the cumulative oxygen transfer after the first maximum is alike for *T.* *reesei* and *P.* *verruculosum*, but much slower for *A.* *terreus*. Furthermore, the overall amount of oxygen consumed and the final cellulose concentration are quite similar in all cultures. Therefore, it can be concluded that the cumulative oxygen consumption of the organisms is correlated to the total amount of cellulose consumed.

During the consumption of cellulose, the pH of the culture decreases according to Fig. 2b. The higher rate of cellulose consumption for *T. reesei* and *P. verruculosum* results in a steeper pH drop within the first 72 h. Afterwards, the pH remains constant for *T. reesei* and increases slightly for *P. verruculosum* reaching a pH of 3.6 or 4.0, respectively. The pH of the *A. terreus* culture slowly declines throughout the cultivation to a pH of 4.2.

The protein content of the culture supernatants and the results of the conventional method to measure cellulase activity by the standard filter paper assay are depicted in Fig. 2d, f. The protein content in the cultures increases during the second increase in OTR. A protein content of about 2400 and 2100 mg L^−1^ was achieved for *T. reesei* and *P.* *verruculosum*, respectively. The final standard filter paper activity was as well slightly higher for the *T. reesei* culture. The final protein content and standard filter paper activity of the *A.* *terreus* culture were several times smaller reaching only 500 mg L^−1^ and 0.2 FPU mL^−1^.

To elucidate which candidate is more suitable for the mixed culture application and to compare the results to the conventional cellulase activity measurement, the samples were also analyzed by the here introduced freeze assay.

**Fig. 3:**
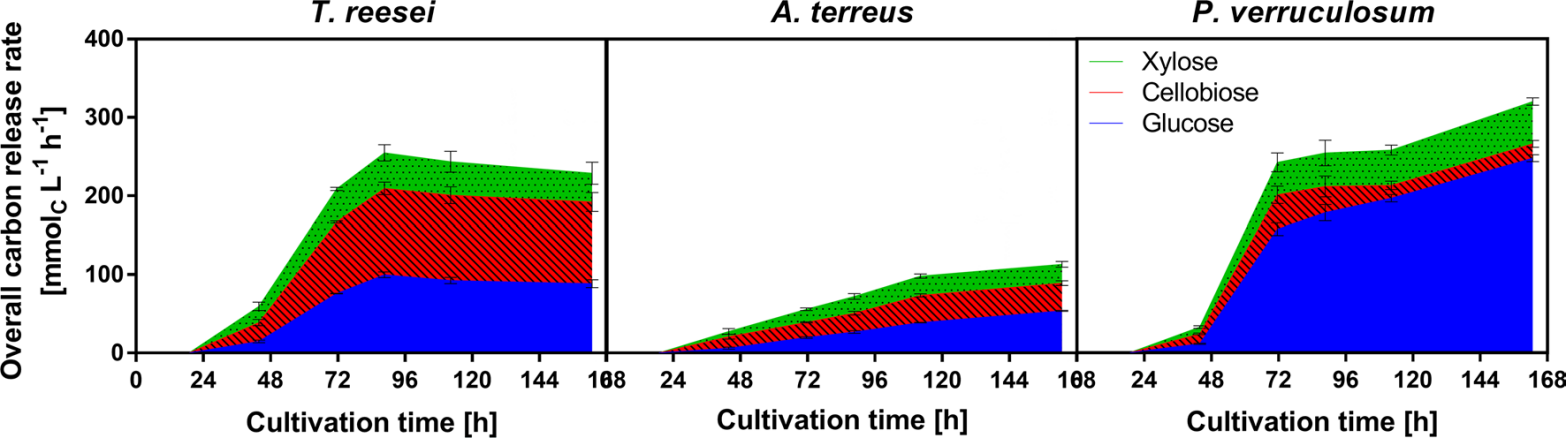
Freeze assay measurements for the cultivations of *T.* *reesei, A.* *terreus*, and *P.* *verruculosum*. Overall carbon release rate as a sum of glucose, cellobiose, and xylose carbon release rate. *Colors* indicate the distribution of sugars. *Error bars* represent standard deviation of technical triplicates. Assay conditions: 91 mM itaconic acid buffer (pH 3.7), 120 g L^−1^ α-cellulose, incubation time 2 h, filling volume 1.1 in 2-mL tube, shaking frequency 900 rpm, shaking diameter 3 mm, and 30 °C

As shown in Fig. 3, CRR_Freeze_ increased continuously during the cultivation of *A. terreus* and *P.* *verruculosum* reaching an overall carbon release rate of 120 and 340 mmol_C_ L^−1^ h^−1^, respectively. In contrast to the results of the standard filter paper activity assay, the maximum CRR_Freeze_ of *T. reesei* was 270 mmol_C_ L^−1^ h^−1^ and, therefore, lower compared to *P.* *verruculosum*. Furthermore, in the *T. reesei* sample much more cellobiose was produced confirming the deficiency in β-glucosidase production. Consequently, based on the freeze assay measurements under target process conditions, *P.* *verruculosum* is the more potent CBP candidate. In contrast, the standard filter paper assay depicted *T.* *reesei* to be the best candidate. However, the assay is not suited to evaluate cellulase producers for CBP applications as the hydrolysis is not performed under fermentation conditions and cell or cellulose bound enzymes are not considered.

### Comparison of the overall carbon release rate of *T. reesei* based on the oxygen transfer rate and freeze assay measurements

The freeze assay measurements and the RAMOS technology were applied to evaluate the suitability of different CBP candidate organisms. To emphasize the differences as well as opportunities and pitfalls of each technique, the results of both methods were compared exemplarily for the cultivation of *T. reesei*.

**Fig. 4:**
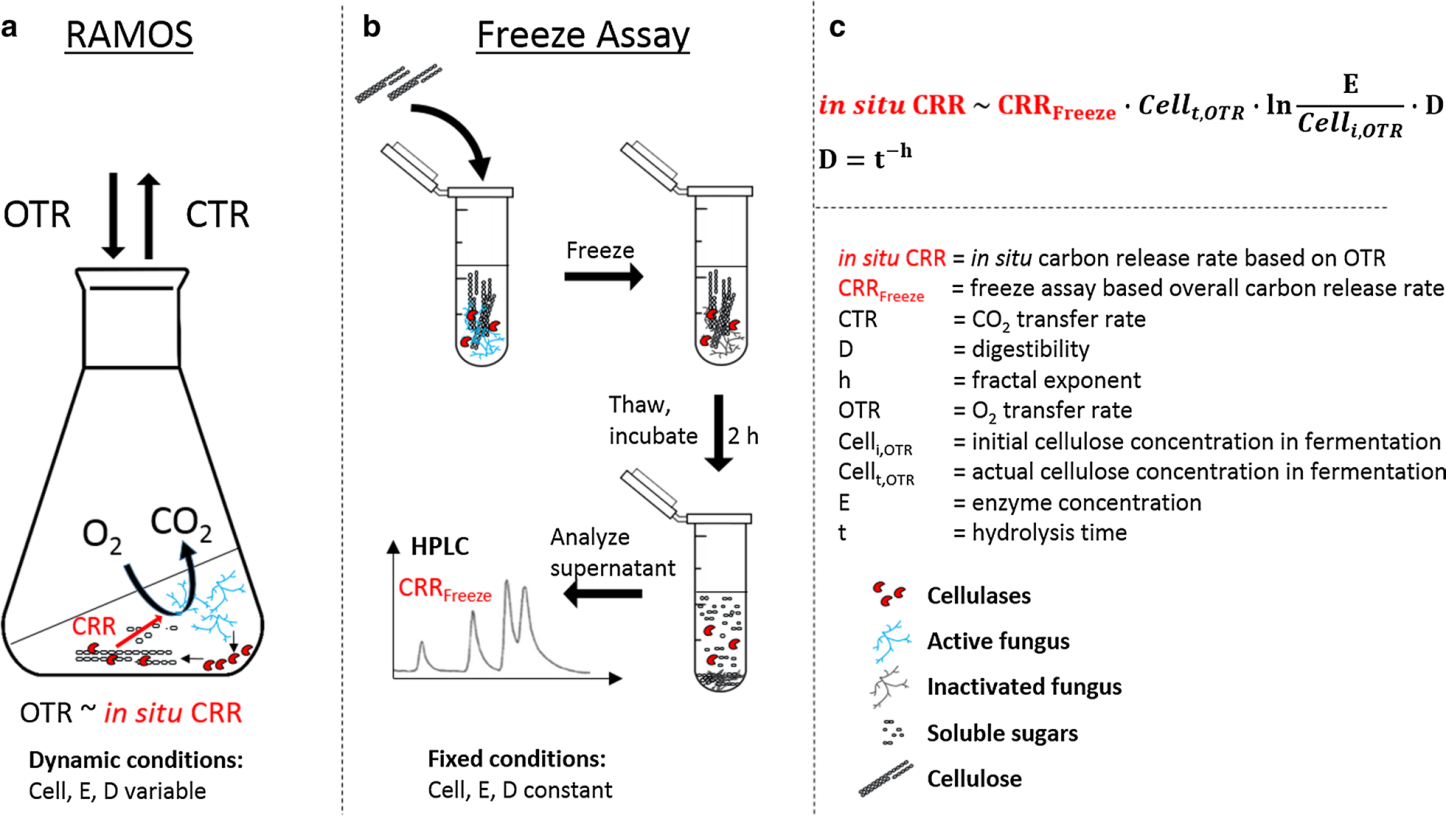
Method scheme: comparison of OTR-based cellulase activity measurement with freeze assay. **a** The RAMOS technique allows the quantification of the released sugars based on the oxygen consumption rate (OTR) during growth on cellulose. The fungus produces cellulases to degrade cellulose into soluble sugars. The sugar and oxygen uptake are assumed to be stoichiometrically coupled. As the conversion of cellulose is the process-limiting step, no monomeric sugars accumulate. Based on the total amount of oxygen consumed, the amount of sugars released can be estimated. **b** The freeze assay allows the quantification of the initial carbon release rate (CRR_Freeze_) under target process conditions. A sample is mixed with a fixed amount of substrate and 1 M itaconic acid buffer at pH 3.7, and then frozen to inactivate the fungus. Then the sample is thawed and incubated for 2 h at fermentation temperature to mimic the in situ conditions. Finally, the supernatant is analyzed by HPLC for soluble sugars to quantify the initial CRR_Freeze_. **c** The in situ CRR is dependent on the actual cellulose concentration (Cell_t_), the enzyme concentration, **e** and cellulose digestibility **d**. These factors change dynamically over the cultivation time: new enzyme is produced, the cellulose is consumed, and the digestibility decreases due to various effects. To compare different fungi under equal conditions, the freeze assay is performed. Because the hydrolysis time is short in comparison to the cultivation time and a defined amount of fresh cellulose is added, the influencing factors cellulose concentration, enzyme concentration, and digestibility can be considered constant. Thus, the freeze assay quantifies the initial hydrolysis rate (CRR_Freeze_) under target process conditions at high cellulose loading. To compare the results of both methods, the CRR_Freeze_ can be transformed into in situ CRR by incorporating the difference in initial cellulose concentration in the fermentation and the freeze assay by a logarithmic correlation of the specific carbon release rate with initial enzyme/cellulose ratio (see Fig. 5b). The decrease in digestibility is included using a fractal kinetic model (see Fig. 5c)

**Fig. 5:**
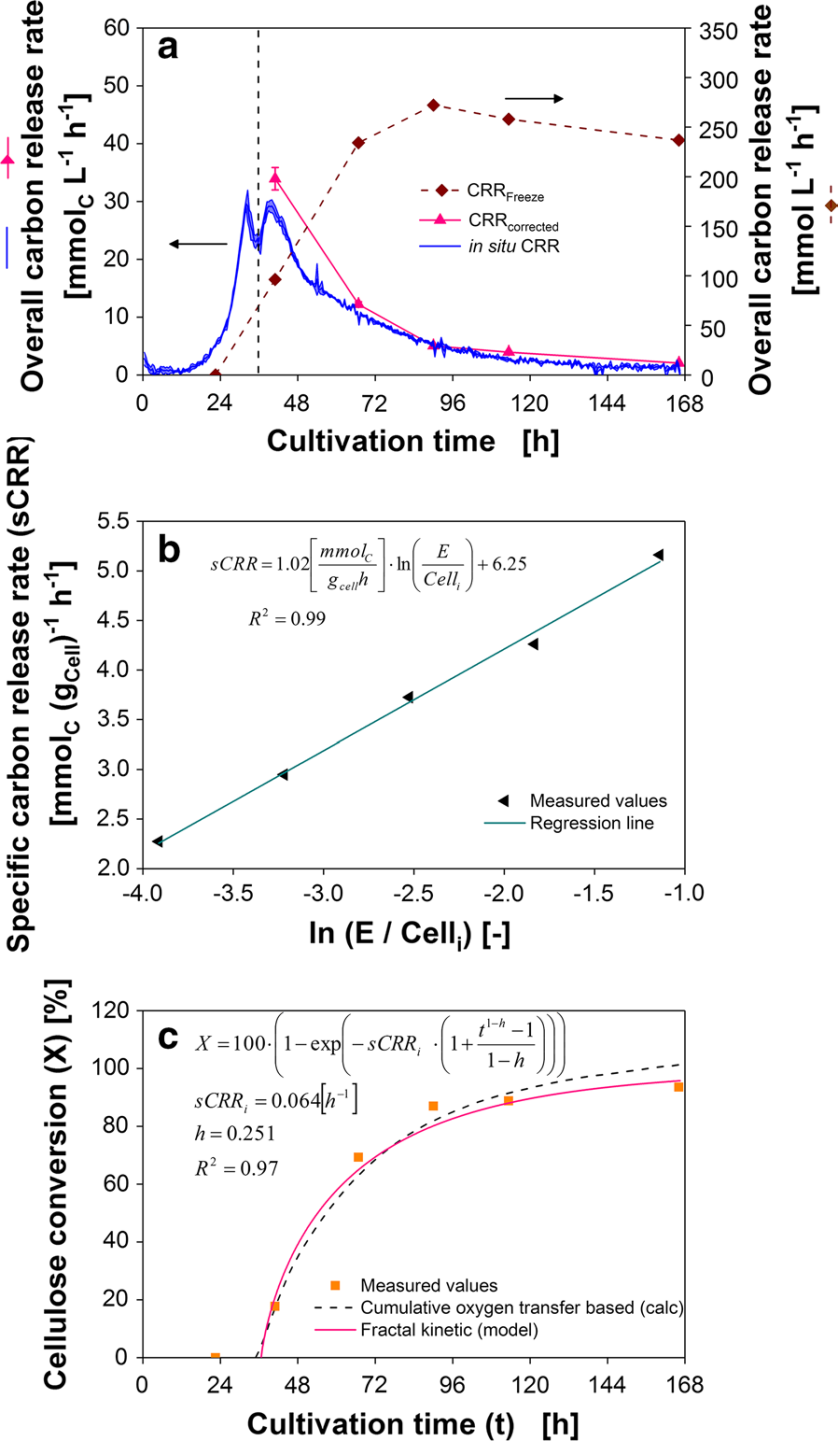
Overall carbon release rate of *T. reesei* based on OTR and freeze assay measurements. **a** Overall carbon release rate calculated from the oxygen transfer rate and freeze assay measurements of the suspended full culture broth. For comparison of the two methods, the measured freeze assay carbon release rate was adapted to the initial substrate concentration in the fermentation broth and modified to account for the decrease in substrate digestibility over cultivation time using a fractal kinetic model. **b** Correlation of specific carbon release rate (sCRR) with initial enzyme/cellulose (E/Cell_i_) ratio for *T.* *reesei*. The carbon release rate was calculated as a sum of glucose, cellobiose, and xylose carbon release rate of the suspended full culture broth during freeze assay. Assay conditions: 91 mM itaconic acid buffer (pH 3.7), 120 g L^−1^ α-cellulose, incubation time 2 h, filling volume 1.1 in 2-mL test tube, shaking frequency 900 rpm, shaking diameter 3 mm, and 30 °C. **c** Cellulose conversion (X) calculated based on the residual cellulose concentration and fractal kinetic model for a cultivation of *T.* *reesei*. sCRR_i_ initial specific freeze assay carbon release rate; h, fractal exponent describing the drop in sCRR_i_ over time

Figure 4a illustrates the employed principle of the RAMOS technology. During growth on cellulose, the fungi produce cellulases to degrade cellulose into soluble sugars. The sugars are taken up and oxygen is consumed. As the rate of hydrolysis is slower than the sugar uptake, no sugars accumulate in this test. This was confirmed by HPLC analysis. Hence, the sugar and oxygen uptake are assumed to be stoichiometrically coupled. Consequently, the measured OTR is proportional to the in situ CRR. The proportionality constant is the molar ratio of the amount of oxygen taken up during growth on cellulose and the amount of cellulose consumed (see Eq. 1). The in situ CRR is depicted in Fig. 5a. The start of cellulose consumption is indicated by a dashed line. The procedure of the freeze assay is shown in Fig. 4b. The released sugars (glucose, cellobiose, and xylose) are measured by HPLC and CRR_Freeze_ is shown in Fig. 5a.

The shape of the OTR-based in situ CRR and freeze assay-based CRR_Freeze_, illustrated in Fig. 5a, are very different. The in situ CRR decreases over the last 5 days of cultivation. In contrast, CRR_Freeze_ resemble a saturation curve. The in situ CRR during cellulose degradation depends on the available cellulose content, the present enzyme concentration, and the cellulose digestibility (Fig. 4c) [38]. The enzyme concentration in the cultivation and the corresponding freeze assay sample is equal but the substrate concentration in the freeze assay is increased by adding 120 g L^−1^ cellulose. To account for this difference, the CRR_Freeze_ was referenced to the cellulose concentration in the fermentation broth. This was achieved by using the excellent correlation (*R* ^2^ = 0.99) between the cellulose-related carbon release rate (named specific carbon release rate, sCRR) and the initial enzyme/cellulose (E/Cell_i_) ratio (Fig. 5b). Furthermore, there is a difference in cellulose degradation time between the two measurements. The cultivation in the RAMOS device lasts for 168 h compared to 2 h cellulose hydrolysis during the freeze assay. As cellulose digestibility decreases over time, this effect has to be considered for the freeze assay [39]. Wang and Feng used a fractal kinetic model to describe the decrease in the cellulose hydrolysis rate during the enzymatic saccharification [40]. The fractal kinetic model employs two parameters, namely the rate coefficient and the fractal exponent. Applied on the present case, the fractal exponent (h) specifies the decrease of the initial specific carbon release rate (sCRR_i_) over cultivation time. The fractal exponent can be calculated based on the course of cellulose conversion over degradation time. Therefore, the curve of cellulose conversion over cultivation time, calculated from the cumulative oxygen transfer, was used to fit the fractal exponent. A high coefficient of correlation of 0.97 was achieved. The results are presented in Fig. 5c. Afterwards, the fractal exponent was integrated to calculate the freeze assay-based overall carbon release rate corrected by the initial enzyme/cellulose ratio in the fermentation and by fractal kinetic (CRR_corrected_). The resulting curve, depicted in Fig. 5a, is in good agreement with the in situ CRR.

The two main differences influencing the CRR_Freeze_ and in situ CRR were identified and give insight into the scope of application for both methods. These differences are the present cellulose concentration and the deviating cellulose digestibility. Therefore, it has to be noticed that CRR_Freeze_ overestimates the actual carbon release rate due to the decrease of cellulose digestibility over time, which is not taken into account by CRR_Freeze_. However, using the presented method to calculate the fractal exponent, the decrease in cellulose digestibility can be incorporated.

The in situ CRR allows to assess the cellulase induction time and to estimate the cellulase production rate by evaluating the second increase in OTR. However, after reaching the maximum OTR, the amount of cellulose binding sites becomes limiting. Despite the production of cellulases, the hydrolysis rate decreases and, therefore, the in situ CRR decreases. Thus, it is not possible to conclude on the full enzymatic potential of the culture broth under these dynamic conditions, where available cellulose binding sites are limiting the reaction. The full potential of the culture broth at this stage of cultivation can be assessed by performing the freeze assay.

**Fig. 6:**
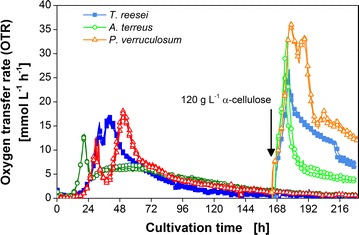
Addition of α-cellulose to cultures of *T.* *reesei, A.* *terreus*, and *P.* *verruculosum*. After 7 days of cultivation, additional 120 g L^−1^ α-cellulose was supplemented to two of the biological triplicates (indicated by an *arrow*). After addition, the OTR is marked with a *lighter corresponding color*. For clarity of the depicted values, only every fifth measuring point is represented by a *symbol*. Culture conditions: modified Pakula medium with 5 g L^−1^ glucose and 30 g L^−1^ α-cellulose, 250-mL flask, filling volume 20 mL, shaking frequency 350 rpm, shaking diameter 50 mm, inoculum 10^6^ spores mL^−1^, and 30 °C

To confirm the findings that the amount of cellulose and its digestibility are the main differences causing discrepancies between the in situ CRR and CRR_Freeze_, parallel cultures of *T.* *reesei*, *A.* *terreus*, and *P.* *verruculosum* shown in Fig. 2 were spiked with 120 g L^−1^ fresh α-cellulose after 168 h of cultivation. As seen in Fig. 6, the OTR of all cultures is highly reproducible and increases immediately to 10 mmol L^−1^ h^−1^ after the addition of cellulose. Afterwards, the OTR reaches 27 to 37 mmol L^−1^ h^−1^ for the different organisms. Due to the high enzyme and cellulose concentration, it can be assumed that immediately after the spike soluble sugars temporarily accumulate. Therefore, it is not the hydrolysis rate that limits the respiration activity at this stage, but the respiration capacity of the organisms is probably the bottleneck of the process. After 178 h, the OTR suddenly drops reaching different plateaus for each organism. The sudden drop in OTR is probably caused by the emerging limitation of soluble sugars caused by the depletion of the surplus sugars from the initial fast hydrolysis. From that point on, the OTR is likely again determined by the actual hydrolysis rate. The different plateaus of the OTR suggest *P.* *verruculosum* to have the highest in situ CRR, followed by *T. reesei* and *A. terreus*. These results support the assessment of the freeze assay measurements, suggesting *P. verruculosum* as the most suitable candidate for the targeted CBP application. To complete the setup of the targeted CBP application, the interactions between the organisms and the effect of their metabolic products on each other have to be elucidated. This issue is currently under investigation.

## Conclusions

Two complementing methods are presented to evaluate the cellulolytic potential of cellulase-producing organisms for a mixed culture application to convert cellulose into a valuable product. The introduced freeze assay, performed under targeted consolidated bioprocessing (CBP) conditions, allows for a fast assessment of the initial carbon release rate under different conditions. To further characterize the most promising cellulase producers, a second method, measuring the oxygen transfer rate (OTR) and converting this signal into the in situ CRR of the culture during its growth on cellulose, was applied. To demonstrate the potential of the methods, both were used to identify the best cellulolytic partner for a CBP mixed culture system to convert cellulose into the platform chemical itaconic acid. Five different cellulase-producing fungi were compared based on the results of the freeze assay. *T.* *reesei* and *P.* *verruculosum* were the most promising candidates and therefore deeper investigated. The observed OTR profiles revealed that cellulase production was induced faster in *T.* *reesei* and the itaconic acid producer *A.* *terreus* compared to *P.* *verruculosum*. The initial cellulase production rate, indicated by the increase in OTR during cellulose utilization, was similar for *T.* *reesei* and *P.* *verruculosum* and much higher compared to *A.* *terreus*. The carbon release rate measured by OTR and freeze assay was compared and an identified discrepancy could be explained by the different initial enzyme/cellulose ratio as well as the different cellulose digestibility. Finally, both methods suggested *P.* *verruculosum* to be the most suitable candidate for the investigated CBP application, performing slightly better than *T. reesei*. The prospective potential of the applied *P.* *verruculosum* is striking, especially due to the comparison to the benchmark organism *T. reesei* Rut-C30, which was adapted to high cellulase production for over 30 years by several rounds of mutagenesis. This example clearly demonstrates the potential of the presented methods for future design of consolidated bioprocesses.

## Methods

### Microorganisms

In this study, five different fungi were investigated: *T. reesei* Rut-C30 (ATCC 56765), *A. terreus* (DSM 23081), *P. verruculosum* M28-10 (DSM 8069), *M. thermophila* (DSM 1799), and *T. terrestris* (CBS 351.90). The spore suspensions were prepared using malt extract agar medium (Sigma-Aldrich, St. Louis, USA) for *P. verruculosum* M28-10, *M.* *thermophila, and T.* *terrestris*, potato extract glucose agar medium (Roth, Karlsruhe, Germany) for *T. reesei* Rut-C30, and Czapek-Dox agar medium for *A. terreus*. The Czapek-Dox agar medium contained sucrose 30 g L^−1^, NaNO_3_ 3 g L^−1^, KCl 0.5 g L^−1^, MgSO_4_·7H_2_O 0.5 g L^−1^, FeSO_4_·7H_2_O 0.01 g L^−1^, K_2_HPO_4_ 1 g L^−1^, and agar 13 g L^−1^. The pH was set to 7.2 with 1 M H_2_SO_4_. The plates were incubated for 10–14 days at 37 °C for *M.* *thermophila* and *T.* *terrestris* or 30 °C for the remaining fungi until sporulation occurred. The agar plates were harvested using 10 mL 0.9% (w/v) sodium chloride solution. The spore concentration was determined in a Neubauer-Improved counting chamber (Superior Marienfeld, Lauda-Königshofen, Germany). The solution was stored for up to four weeks at 4 °C until inoculation. For cryopreservation at −80 °C, the spore suspension was mixed with glycerol to yield 20% (v/v).

### Cultivation conditions

The preliminary cellulase activity screening was conducted in 500-mL non-baffled shake flasks with a filling volume of 50 mL. The culture was inoculated with 10% (v/v) of a homogenized pre-culture. 45 mL culture broth in a 50-mL plastic tube was homogenized using an Ultra-Turrax^®^ T10 standard (IKA^®^-Werke GmbH & Co. KG, Staufen, Germany) equipped with the dispersion tool S10 D-7G-KS-110 at level 2 for 1 min. The flasks were incubated in an orbital shaker with a shaking frequency of 200 rpm and a shaking diameter of 25 mm.

The remaining experiments were performed with a filling volume of 20 mL in 250-mL non-baffled shake flasks, while shaking at 350 rpm with a shaking diameter of 50 mm. The culture was either inoculated with 10% (v/v) of a pre-culture for the general assessment of the cellulase producers or with a spore suspension to a final concentration of 10^6^ spores mL^−1^. The respective cultivation temperature is stated in the caption of the figures.

### Media and solutions

The cultivations were performed in a modified Pakula medium [41]. The medium consists of (NH_4_)_2_SO_4_ 7.6 g L^−1^, KH_2_PO_4_ 2.6 g L^−1^, MgSO_4_·7H_2_O 0.5 g L^−1^, CaCl_2_·2H_2_O 0.23 g L^−1^, NaCl 0.05 g L^−1^, 1,4-Piperazinedipropanesulfonic acid (PIPPS) 33 g L^−1^ (0.1 M), glucose 5 g L^−1^, urea 0.3 g L^−1^, peptone ex casein 2 g L^−1^ (Roth, Karlsruhe, Germany), tween 80 0.1% (v/v), trace element solution 2.5 mL L^−1^. The pH of the medium without trace elements and cellulose was set to 5.5 with 5 M NaOH. The trace element solution has the following composition: citric acid 180 g L^−1^, Fe_2_(SO_4_)_3_ 2.29 g L^−1^, ZnSO_4_·7H_2_O 16 g L^−1^, CuSO_4_ 2.05 g L^−1^, MnSO_4_·7H_2_O 1.6 g L^−1^, H_3_BO_3_ 0.8 g L^−1^, CoCl_2_·6H_2_O 2.71 g L^−1^. The necessary amount of cellulose was directly weighted into empty shake flasks (375 mg avicel for the preliminary screening and 600 mg α-cellulose for the remaining experiments) and heat-sterilized as powder before the liquid medium was added. In order to add cellulose during the cultivation, 2.4 g α-cellulose was heat-sterilized as powder and added separately to each shake flask. Both types of cellulose were purchased from Sigma-Aldrich (St. Louis, USA). Glucose and PIPPS were omitted in the preliminary screening. All chemicals were of analytical grade and the solutions were sterile-filtered using 0.2-µm cut-off filters.

### Respiration activity monitoring system (RAMOS)

The respiration activity was measured by an in-house build respiration activity monitoring system (RAMOS). The device is equipped with eight flasks with oxygen partial pressure sensors and differential pressure sensors in order to calculate the oxygen transfer rate (OTR) and the carbon dioxide transfer rate (CTR) [42, 43]. A commercial version of the device can be obtained from Kühner AG (Birsfelden, Switzerland) or HiTec Zang GmbH (Herzogenrath, Germany). Experiments in RAMOS flasks were performed without interruption for taking samples to avoid disruption of the measurement.

### Sample analytics

During the initial cellulase activity screening, samples were taken after 3, 6, and 10 days of cultivation from the same shake flasks. For the experiments with the RAMOS device, cotton plug-sealed shake flasks, ran in parallel to the RAMOS flasks under identical culture conditions, were harvested for offline analysis. All flasks were inoculated from the same master mix. To obtain enough sample volume, culture broths from two biological duplicates were pooled. The pH of full culture broth was measured with a CyberScan pH 510 device (Eutech Instruments, Landsmeer, The Netherlands). To determine the cellulose concentration, 10 mL full culture broth was transferred to a weighted plastic tube and centrifuged at 3130*g* for 15 min at 4 °C. After removing the supernatant, the pellet was analyzed according to the method of Updegraff [44] adapted by Ahamed and Vermette [45]. Thereby, the fungal biomass is selectively removed by acidic hydrolysis and the remaining cellulose is gravimetrically measured. Protein concentration of the culture supernatant was determined by a Bradford assay [46] using Bradford Reagent (Sigma-Aldrich, St. Louis, USA) and bovine serum albumin as standard.

### Filter paper activity

Cellulase activity in the culture supernatant was measured by the standard filter paper activity (FPA) assay according to the method of Ghose [47] adapted by Xiao [4]. The assay was performed in 96 µL reaction volume in 96-well microtiter plates. 0.1 M sodium acetate buffer (pH 4.8) was used for buffering. The incubation step took place in a conditioned water bath at 50 °C for 1 h. The produced reducing sugars were analyzed by the p-hydroxybenzoic acid hydrazide (PAHBAH) assay with glucose as a standard [48]. The used procedure is previously published by Antonov et al. [12]. Each sample was measured in at least two dilutions each as triplicate.

### Freeze assay activity

1 mL of full culture broth with cells or culture supernatant was added to a 2-mL test tube containing 120 mg α-cellulose and 100 µL of 1 M itaconic acid buffer (pH 3.7). As blank, culture supernatant without α-cellulose was used. The test tubes were frozen at least over night to inactivate the fungi. To verify the complete inactivation of the fungi, a frozen and thawed sample of the culture broth was incubated at the corresponding cultivation temperature for 24 h. No residual respiration activity was detected. After thawing the samples, the test tubes were incubated for 2 h in a thermomixer MKR 13 (DITABIS AG, Pforzheim, Germany) at the respective cultivation temperature. The shaking frequency was 900 rpm at a shaking diameter of 3 mm. After centrifugation of the reaction mixture (16,900*g*; 10 min.; 4 °C) and a second centrifugation step of the resulting supernatant (3000*g*; 10 min.), glucose, cellobiose, and xylose concentration in the supernatant were analyzed by HPLC (Dionex HPLC UltiMate 3000, Thermo Scientific, Waltham, USA) at 65 °C using the following setup: Column: AMINEX Ion Exclusion HPX-87H, 300  ×  7.8 mm (Bio-Rad Laboratories GmbH, Munich, Germany); detectors: Dionex™ Ultimate 3000 UV/VIS detector (Thermo Scientific, Waltham, USA) at 210 nm and RI-101 refractory index detector (Shodex, Munich, Germany); mobile phase: 5 mM sulfuric acid; flow rate: 0.7 mL min^−1^. To evaluate the release of different sugar constituents from the cellulose in a comparable way, the freeze assay-based overall carbon release rate (CRR_Freeze_) in $\left[ {\frac{{{\text{mmol}}_{\text{C}} }}{\text{L h}}} \right]$ was calculated using the molar amount of carbon released in form of glucose, cellobiose, and xylose.

### Respiration activity-based calculations

The in situ carbon release rate (in situ CRR) was calculated based on Eq. (1) for growth on cellulose.

1 $$\text{in situ} \,\text{CRR} = \frac{{{\text{OTR}} \cdot \text{A}_{\text{Cell}} }}{{\text{Y}_{{{\text{O}}_{2} /{\text{Cell}}}} }} \left[ {\frac{{{\text{mmol}}_{\text{C}} }}{\text{L h}}} \right],$$

where OTR is the oxygen transfer rate, A_Cell_ is the number of carbon atoms in the cellulose repeating unit, and ${\text{Y}}_{{{\text{O}}_{ 2} /{\text{Cell}}}}$ is the molar ratio of the molar amount of oxygen taken up during growth on cellulose divided by the molar amount of cellulose consumed. The molar amount of cellulose was calculated using the molecular weight of the cellulose repeating unit. The parameters are listed in Table 2. Error area for in situ CRR represents the standard deviation of biological triplicates.

**Table 2:** Calculation parameters

Variable	Meaning	Value	Unit
Y_O2/Cell_	Molar ratio of oxygen and cellulose	3.39	mol mol^−1^
A_Cell_	Number of carbon atoms in cellulose repeating unit	6	–
MW_Cell_	Molecular weight of cellulose repeating unit	162.14	g mol^−1^

### Freeze assay-based calculations

To compare the OTR-based in situ CRR and freeze assay-based CRR_Freeze_, a two-step procedure was applied.

In the first step, CRR_Freeze_ was adapted to the same initial enzyme/cellulose (E/Cell_i,OTR_) ratio as in the fermentation. To achieve this, the following general correlation between the specific carbon release rate sCRR (defined as carbon release rate CRR divided by the initial cellulose concentration Cell_i_) and the natural logarithm of E/Cell_i_ ratio was used:

2 $$\frac{\text{CRR}}{{{\text{Cell}}_{\text{i}} }} = {\text{sCRR}} = \text{m} \cdot \ln \left( {\frac{\text{E}}{{{\text{Cell}}_{\text{i}} }}} \right) + \text{b} \left[ {\frac{{{\text{mmol}}_{\text{C}} }}{\text g\; \text h}} \right].$$

The calibration curve used to calculate the slope $\left( {\text{m} = 1.02 \left[ {\frac{{{\text{mmol}}_{\text{C}} }}{\text g \;\text h}} \right]} \right)$ and the axis intercept $\left( {\text{b} = 6.25 \left[ {\frac{{{\text{mmol}}_{\text{C}} }}{\text g\; \text h}} \right]} \right)$ is shown in Fig. 5b.

As the initial cellulose concentration in the fermentation is known, only the enzyme concentration is needed for the transformation. To calculate the enzyme concentration (E) present in a sample, the measured CRR_Freeze_ was used applying Eq. 2:

3 $$\frac{{{\text{CRR}}_{\text{Freeze}} }}{{{\text{Cell}}_{{\text{i},{\text{Freeze}}}} }} = \text{m} \cdot { \ln }\left( {\frac{\text{E}}{{{\text{Cell}}_{{\text{i},{\text{Freeze}}}} }}} \right) + \text{b} \left[ {\frac{{{\text{mmol}}_{\text{C}} }}{\text g\; \text h}} \right],$$

where Cell_i,Freeze_ is the initial cellulose concentration in the freeze assay.

After rearranging Eq. 3 to

4 $$\text{E} = {\text{Cell}}_{{\text{i},{\text{Freeze}}}} \cdot \exp \left( {\frac{{\frac{{{\text{CRR}}_{\text{Freeze}} }}{{{\text{Cell}}_{{\text{i},{\text{Freeze}}}} }} - \text{b}}}{\text{m}}} \right) \left[ {\frac{\text{g}}{\text{L}}} \right]$$

and inserting Eq. 4 into Eq. 2, the freeze assay-based specific carbon release rate corrected by the E/Cell_i,OTR_ ratio is given by Eq. 5.

5 $${\text{sCRR}}_{{\text{E}/{\text{Cell}}_{{\text{i},{\text{OTR}}}} }} = \text{m} \cdot \ln \left( {\frac{{{\text{Cell}}_{{\text{i},{\text{Freeze}}}} \cdot \exp \left( {\frac{{\frac{{{\text{CRR}}_{\text{Freeze}} }}{{{\text{Cell}}_{{\text{i},{\text{Freeze}}}} }} - \text{b}}}{\text{m}}} \right)}}{{{\text{Cell}}_{{\text{i},{\text{OTR}}}} }}} \right) + \text{b} \left[ {\frac{{{\text{mmol}}_{\text{C}} }}{\text g \;\text h}} \right].$$

In order to calculate the freeze assay-based overall carbon release rate corrected by the E/Cell_i,OTR_ ratio, the equation is rearranged and multiplied with the actual cellulose concentration (Cell_t,OTR_) measured during the fermentation.

6 $${\text{CRR}}_{{\text{E}/{\text{Cell}}_{{\text{i},{\text{OTR}}}} }} = \left( {\text{m} \cdot \ln \left( {\frac{{{\text{Cell}}_{{\text{i},{\text{Freeze}}}} }}{{{\text{Cell}}_{{\text{i},{\text{OTR}}}} }}} \right) + \frac{{{\text{CRR}}_{\text{Freeze}} }}{{{\text{Cell}}_{{\text{i},{\text{Freeze}}}} }}} \right) \cdot {\text{Cell}}_{{\text{t},{\text{OTR }}}} \left[ {\frac{{{\text{mmol}}_{\text{C}} }}{\text{L h}}} \right].$$

The error $\Delta {\text{CRR}}_{{\text{E}/{\text{Cell}}_{{\text{i},{\text{OTR}}}} }}$ was calculated according to Gaussian error propagation, considering the error of the slope of the calibration curve $(\Delta \text{m} = 0.04)$ and the error of CRR_Freeze_ (ΔCRR_Freeze_).

7 $$\Delta {\text{CRR}}_{{\text{E}/{\text{Cell}}_{{\text{i},{\text{OTR}}}} }} = \sqrt {\left( {{ \ln }\left( {\frac{{{\text{Cell}}_{{\text{i},{\text{Freeze}}}} }}{{{\text{Cell}}_{{\text{i},{\text{OTR}}}} }}} \right) \cdot {\text{Cell}}_{{\text{t},{\text{OTR}}}} \cdot \Delta {\text{m}}} \right)^{2} + \left( {\frac{{\Delta {\text{CRR}}_{\text{Freeze}} }}{{{\text{Cell}}_{{\text{i},{\text{Freeze}}}} }} \cdot {\text{Cell}}_{{\text{t},{\text{OTR}}}} } \right)^{2} }.$$

In the second step, the decrease in cellulose digestibility during the fermentation was incorporated into the freeze assay-based overall carbon release rate corrected by the E/Cell_i,OTR_ ratio. This was achieved by applying the fractal kinetic of Wang and Feng [40] using Eq. 8.

8 $${\text{CRR}}_{\text{corrected}} = {\text{CRR}}_{{\text{E}/{\text{Cell}}_{{\text{i},{\text{OTR}}}} }} \cdot \text{t}^{ \text{- h}} \left[ {\frac{{{\text{mmol}}_{\text{C}} }}{{\text{L h}}}} \right],$$

where h ($\text{h} = 0.251$) is the fractal exponent describing the drop in digestibility with time (t).

The following equation from Wang and Feng [40] was applied to fit the fractal exponent

9 $$\text{X} = 100 \cdot \left( {1 - { \exp }\left( { - {\text{sCRR}}_{\text{i}} \cdot \left( {1 + \frac{{t^{1 - \text{h}} - 1}}{1 - \text{h}}} \right)} \right)} \right) \left[{\text{\%}} \right],$$

where X is the degree of cellulose conversion. The initial specific freeze assay carbon release rate ${\text{sCRR}}_{\text{i}} = 0.064\,\text{h}^{ - 1}$ was estimated based on the measured freeze assay activity of the first sample from the *T. reesei* Rut-C30 culture with the start of cellulose digestion.

To fit the fractal exponent, the course of cellulose conversion X(t) was calculated based on the cumulative oxygen transfer and plotted over cultivation time, starting from the moment of cellulose conversion.

10 $$\text{X}(\text{t}) = \frac{{\mathop \smallint \nolimits_{0}^{t} {\text{OTR}} \cdot {\text{MW}}_{\text{Cell}} }}{{\text{Y}_{{{\text{O}}_{2} /{\text{Cell}}}} \cdot {\text{Cell}}_{{\text{i},{\text{OTR}}}} }} \cdot 100 \left[ {\text{\%}} \right],$$

where MW_Cell_ is the molecular weight of the cellulose repeating unit given in Table 2.

## Additional files

**Additional file 1: Figure S1.** Screening for cellulase synergism with *A. terreus* culture broth using the freeze assay. Freeze assay was performed at the indicated cultivation temperatures with 0.5 mL suspended full culture broth of *A. terreus* mixed with 0.5 mL full culture broth of different candidate organisms. Full culture broth was harvested after 5 days of cultivation in modified Pakula medium with 5 g L^−1^ glucose and 30 g L^−1^ α-cellulose. *Bars* show overall carbon release rate calculated as a sum of glucose, cellobiose and xylose carbon release rate. *Colors* indicate the distribution of sugars. *Error bars* show standard deviation from triplicates. AT = *Aspergillus terreus,* TR = *Trichoderma reesei*, PV = *Penicillium verruculosum*, MT = *Myceliophtora thermophila.* Assay conditions: 91 mM itaconic acid buffer (pH 3.7), 120 g L^−1^ α-cellulose, incubation time 2 h, filling volume 1.1  mL in 2 mL test tube, shaking frequency 900 rpm, shaking diameter 3 mm.

**Additional file 2: Figure S2.** Characteristic growth and enzyme production properties of *T. terrestris.* **(A)** Biological duplicates of oxygen transfer rate (OTR) and cumulative oxygen transfer. For clarity of the depicted values, only every third measuring point of OTR and every eighth measuring point of cumulative oxygen transfer over time is represented by a *symbol*. **(B)** pH value and protein content in the culture supernatant; **(C)** Residual cellulose concentration and standard filter paper activity; RAMOS flasks were cultivated for OTR assessment without interruption. For offline analysis in B and C shake flasks run in parallel, inoculated from the same master mix, were harvested. *Error bars* represent standard deviation of technical triplicates from pooled biological duplicates. Culture conditions: modified Pakula medium with 5 g L^−1^ glucose and 30 g L^−1^ α-cellulose, 250 mL flask, filling volume 20 mL, shaking frequency 350 rpm, shaking diameter 50 mm, inoculum 10^6^ spores mL^−1^, and 37 °C.

## Abbreviations

b: axis intercept
A_Cell_: number of carbon atoms in the cellulose repeating unit
CBP: consolidated bioprocessing
Cell: cellulose concentration
Cell_i,Freeze_: initial cellulose concentration in freeze assay
Cell_i,OTR_: initial cellulose concentration in fermentation
Cell_t,OTR_: actual cellulose concentration in fermentation
${\text{CRR}}_{{\text{E}/{\text{Cell}}_{{\text{i} , {\text{OTR}}}} }}$: freeze assay-based overall carbon release rate corrected by the initial enzyme/cellulose ratio in the fermentation
CRR_corrected_: freeze assay-based overall carbon release rate corrected by the initial enzyme/cellulose ratio in the fermentation and fractal kinetic
CRR_Freeze_: freeze assay-based overall carbon release rate
E: enzyme concentration
FPA: filter paper activity
FPU: filter paper unit
Glc: glucose
h: fractal exponent
in situ CRR: OTR-based in situ carbon release rate
m: slope
MW: molecular weight
OTR: oxygen transfer rate
PAHBAH: p-hydroxybenzoic acid hydrazide
RAMOS: respiration activity monitoring system
sCRR: specific carbon release rate
sCRR_i_: initial specific freeze assay carbon release rate
${\text{sCRR}}_{{\text{E}/{\text{Cell}}_{{\text{i},{\text{OTR}}}} }}$: freeze assay-based specific carbon release rate corrected by the initial enzyme/cellulose ratio in the fermentation
t: hydrolysis time
X: cellulose conversion
$\text{Y}_{{{\text{O}}_{ 2} / {\text{Cell}}}}$: molar ratio of the molar amount of oxygen taken up during growth on cellulose divided by the molar amount of cellulose consumed
