# Supplementary figures and images for: Process relevant screening of cellulolytic organisms for consolidated bioprocessing
Source: Biotechnol Biofuels. 2017 Apr 24;10:106. doi: 10.1186/s13068-017-0790-4 (PMC5402656; doi:10.1186/s13068-017-0790-4)

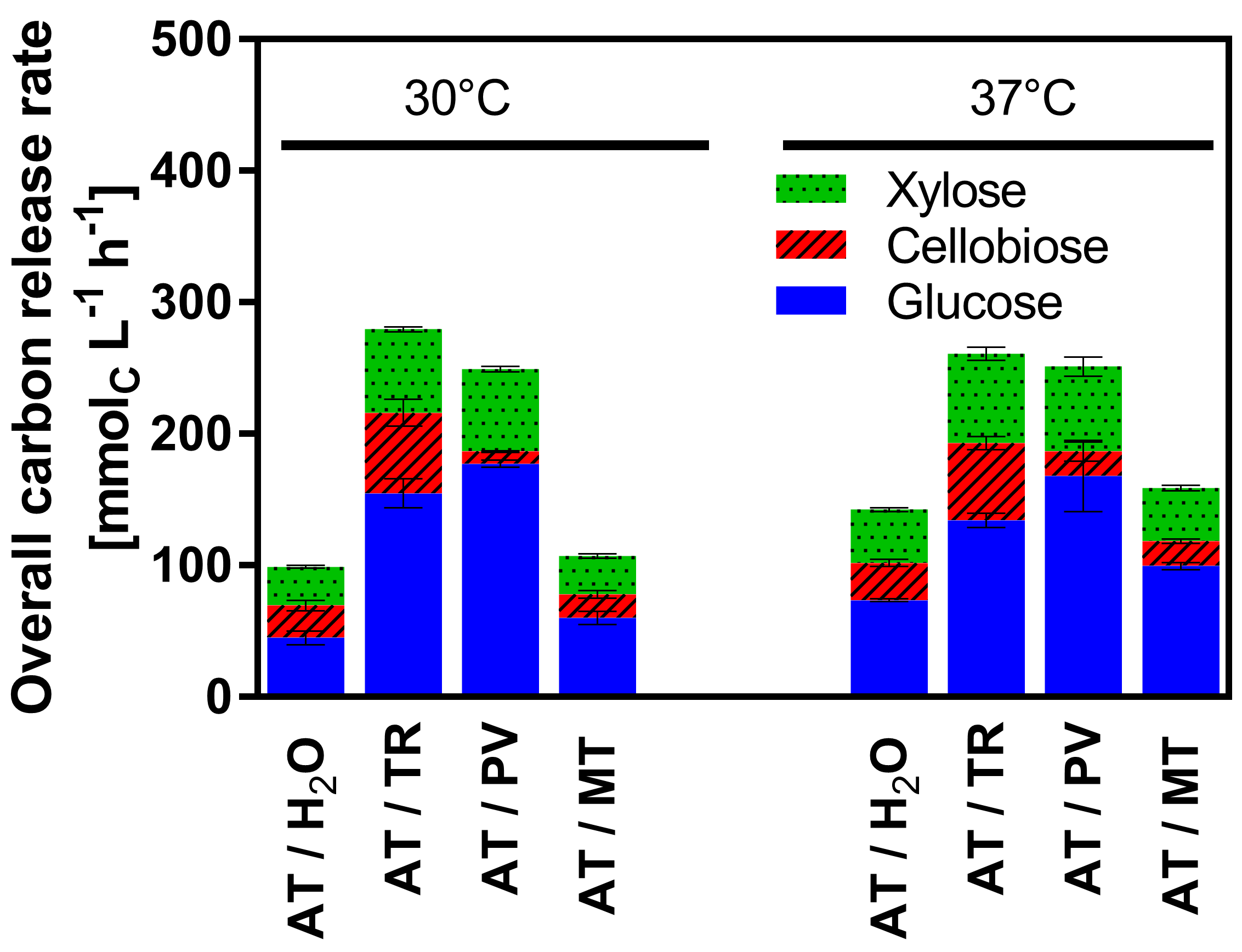

Supplement: Supplementary file 1 — Additional file 1: Figure S1. Screening for cellulase synergism with A. terreus culture broth using the freeze assay. Freeze assay was performed at the indicated cultivation temperatures with 0.5 mL suspended full culture broth of A. terreus mixed with 0.5 mL full culture broth of different candidate organisms. Full culture broth was harvested after 5 days of cultivation in modified Pakula medium with 5 g L−1 glucose and 30 g L−1 α-cellulose. Bars show overall carbon release rate calculated as a sum of glucose, cellobiose and xylose carbon release rate. Colors indicate the distribution of sugars. Error bars show standard deviation from triplicates. AT = Aspergillus terreus, TR = Trichoderma reesei, PV = Penicillium verruculosum, MT = Myceliophtora thermophila. Assay conditions: 91 mM itaconic acid buffer (pH 3.7), 120 g L−1 α-cellulose, incubation time 2 h, filling volume 1.1 mL in 2 mL test tube, shaking frequency 900 rpm, shaking diameter 3 mm. [file 13068_2017_790_MOESM1_ESM.tif]
